# Retraction: Comparison of Oral versus Intravenous Proton Pump Inhibitors in Preventing Re-bleeding from Peptic Ulcer after Successful Endoscopic Therapy

**DOI:** 10.7759/cureus.r33

**Published:** 2021-06-14

**Authors:** Rashid Karim, Rizwan Hameed, Kashif Ali, Amber Tahir

**Affiliations:** 1 Gastroenterology, Dera Ghazi Khan Medical College and Hospital, Dera Ghazi Khan, PAK; 2 Internal Medicine, Dera Ghazi Khan Medical College and Hospital, Dera Ghazi Khan, PAK; 3 Internal Medicine, Dow University of Health Sciences, Karachi, PAK

Cureus was recently alerted to similarities between this article and an article published in the October 2018 issue of the Middle East Journal of Digestive Diseases (MEJDD):

Valizadeh Toosi SM, Elahi Vahed AR, Maleki I, Bari Z. Comparison of Oral versus Intravenous Proton Pump Inhibitors in Preventing Re-bleeding from Peptic Ulcer after Successful Endoscopic Therapy. *Middle East J Dig Dis*. 2018;10(4):236-241. doi:10.15171/mejdd.2018.116 https://www.ncbi.nlm.nih.gov/pmc/articles/PMC6488504/

Upon closer examination, we found that while there is no direct text or data duplication, it is clear that the study design described by Karim et al. was duplicated from the previously published article without any form of citation or acknowledgement. Cureus has attempted to reach out to the authors of this article several times and have received no response. Therefore we have made the decision to retract this article due to plagiarism.

